# Narvin’s Veterinary Practice

**Published:** 1881-10

**Authors:** 


NARVIN'S VETERINARY PRACTICE, OR EXPLANATORY STOCK DOCTOR. Copiously illustrated by cuts and engravings. By JOHN NICHOLSON NARVIN, V.S. Indianapolis: John B. Hann. 1880. 8vo., pp. 730.

The work is divided into two sections; the first devoted exclusively to the horse, the second to cattle, sheep, swine and poultry. The first part is divided into four divisions; the first treating of the diseases of the horse; the second to a brief history of the horse—breeding, training, stables, feeding, etc.; the third to the anatomy and age; the fourth to general remedies. The book is written in common terms, especially for the laity, by whom it will be readily understood. Although not pretending to be a classical and strictly scientific work, it is full of good common sense, and shows the great and varied experience of its author. It unquestionably will be of great service in the field for which it is designed.

There is no mysticism, charlatancy or clap-trap about it, but plain facts are related in a clear and intelligent manner. It is a work which will be of no little help to veterinarians, as well as to those not specially trained in veterinary practice. It is the best work that has yet been written by an American author.

